# Delirium prediction in the intensive care unit: comparison of two delirium prediction models

**DOI:** 10.1186/s13054-018-2037-6

**Published:** 2018-05-05

**Authors:** Annelies Wassenaar, Lisette Schoonhoven, John W. Devlin, Frank M. P. van Haren, Arjen J. C. Slooter, Philippe G. Jorens, Mathieu van der Jagt, Koen S. Simons, Ingrid Egerod, Lisa D. Burry, Albertus Beishuizen, Joaquim Matos, A. Rogier T. Donders, Peter Pickkers, Mark van den Boogaard

**Affiliations:** 1 Department of Intensive Care Medicine, Radboud Institute for Health Sciences, Radboud university medical center, P.O. Box 9101, 6500 HB Nijmegen, the Netherlands; 2 Faculty of Health Sciences and National Institute for Health Research Collaboration for Leadership in Applied Health Research and Care (Wessex), University of Southampton, Southampton, UK; 3 Scientific Institute for Quality of Healthcare, Radboud Institute for Health Sciences, Radboud university medical center, Nijmegen, the Netherlands; 4 School of Pharmacy, Northeastern University, Boston, USA; 5 Division of Pulmonary, Critical Care and Sleep Medicine, Tufts Medical Center, Boston, USA; 6 Intensive Care Unit, The Canberra Hospital, Canberra, Australia; 7 Faculty of Health, University of Canberra, Canberra, Australia; 8 College of Health and Medicine, Australian National University, Canberra, Australia; 9 Department of Intensive Care Medicine and Brain Center Rudolf Magnus, University Medical Centre Utrecht, Utrecht, the Netherlands; 10 Department of Critical Care Medicine, Antwerp University Hospital, University of Antwerp, Edegem, Antwerp, Belgium; 11 Department of Intensive Care, Erasmus Medical Center, Rotterdam, the Netherlands; 12 Department of Intensive Care Medicine, Jeroen Bosch Ziekenhuis, ‘s-Hertogenbosch, the Netherlands; 13 Intensive Care Unit, Rigshospitalet, University of Copenhagen, Copenhagen, Denmark; 14 Leslie Dan Faculty of Pharmacy, University of Toronto, Toronto, Canada; 15 Mount Sinai Hospital, Sinai Health System, Toronto, Canada; 16 Department of Intensive Care, Medisch Spectrum Twente, Enschede, the Netherlands; 17 Department of Intensive Care Medicine, Hospital Espírito Santo, Evora, Portugal; 18 Department for Health Evidence, Radboud Institute for Health Sciences, Radboud university medical center, Nijmegen, the Netherlands; 19 Radboud Center for Infectious Diseases, Radboud Institute for Molecular Life Sciences, Radboud university medical center, Nijmegen, the Netherlands

**Keywords:** Adult, Clinical prediction, Critical illness, Delirium, Intensive care unit

## Abstract

**Background:**

Accurate prediction of delirium in the intensive care unit (ICU) may facilitate efficient use of early preventive strategies and stratification of ICU patients by delirium risk in clinical research, but the optimal delirium prediction model to use is unclear. We compared the predictive performance and user convenience of the prediction  model for delirium (PRE-DELIRIC) and early prediction model for delirium (E-PRE-DELIRIC) in ICU patients and determined the value of a two-stage calculation.

**Methods:**

This 7-country, 11-hospital, prospective cohort study evaluated consecutive adults admitted to the ICU who could be reliably assessed for delirium using the Confusion Assessment Method-ICU or the Intensive Care Delirium Screening Checklist. The predictive performance of the models was measured using the area under the receiver operating characteristic curve. Calibration was assessed graphically. A physician questionnaire evaluated user convenience. For the two-stage calculation we used E-PRE-DELIRIC immediately after ICU admission and updated the prediction using PRE-DELIRIC after 24 h.

**Results:**

In total 2178 patients were included. The area under the receiver operating characteristic curve was significantly greater for PRE-DELIRIC (0.74 (95% confidence interval 0.71–0.76)) compared to E-PRE-DELIRIC (0.68 (95% confidence interval 0.66–0.71)) (*z* score of − 2.73 (*p* < 0.01)). Both models were well-calibrated. The sensitivity improved when using the two-stage calculation in low-risk patients. Compared to PRE-DELIRIC, ICU physicians (n = 68) rated the E-PRE-DELIRIC model more feasible.

**Conclusions:**

While both ICU delirium prediction models have moderate-to-good performance, the PRE-DELIRIC model predicts delirium better. However, ICU physicians rated the user convenience of E-PRE-DELIRIC superior to PRE-DELIRIC. In low-risk patients the delirium prediction further improves after an update with the PRE-DELIRIC model after 24 h.

**Trial registration:**

ClinicalTrials.gov, NCT02518646. Registered on 21 July 2015.

**Electronic supplementary material:**

The online version of this article (10.1186/s13054-018-2037-6) contains supplementary material, which is available to authorized users.

## Background

Delirium, defined as acute brain dysfunction featured by disturbances of attention, awareness, and cognition with a fluctuating course caused by an underlying medical condition [1], occurs frequently in the intensive care unit (ICU), is associated with impaired patient outcome, and substantially increases healthcare costs [2, 3]. Given these deleterious consequences, delirium prevention is crucial.

Delirium preventive measures are important for all ICU patients. However, a delirium prediction model may facilitate early recognition of the patients who may benefit the most from delirium prevention [4]. In the case of limited resources, non-pharmacologic reduction strategies and medication-based strategies may be most relevant for patients who have an increased risk of developing delirium. Prediction models may aid clinical decision making and setting of priorities regarding the use of delirium preventive measures. For instance, when deciding which patient should be admitted to the available room with adequate natural daylight and which patient to the room without it, preferably the patient with the highest delirium risk should be admitted to the room with adequate natural daylight. Also, the use of a delirium prediction model facilitates patient selection for studies on delirium prevention, which is not only efficient in terms of reducing waste, but it may also increase the chance of finding an effect, which ultimately might improve ICU patients’ outcomes. Furthermore, family members can be informed about the patient’s risk of developing delirium and be engaged to help provide strategies to reduce delirium (e.g. cognitive activities) [5]. Involvement of family in patient care in the ICU is stimulated by many ICU societies worldwide [6, 7] and might even increase the prevalence of interventions for delirium prevention and treatment in the ICU [8].

Two delirium prediction models have been validated for use in critically ill adults admitted to the ICU [9–11]. The prediction model for delirium in ICU patients (PRE-DELIRIC model) was developed and validated in a large cohort of Dutch ICU patients [9]. This model, which was recently recalibrated in a multinational cohort [10], reliably predicts ICU patients’ risk of delirium using ten predictors obtained within the first 24 h of ICU admission [10]. However, given that up to 25% of critically ill adults develop delirium within the first 24 h of ICU admission [12, 13], and delirium prevention strategies should be deployed as early as possible, an early prediction model (E-PRE-DELIRIC) was developed to predict the risk of delirium the moment a patient is admitted to the ICU [11]. This E-PRE-DELIRIC model was developed and validated in a multinational cohort and uses nine predictors to predict ICU patients’ risk of delirium [11].

It remains unclear which ICU delirium prediction model might be recommended for daily clinical practice, because the comparative predictive performance of the PRE-DELIRIC and the E-PRE-DELIRIC models and clinicians’ preferences have not been assessed [14]. Therefore, the objective of this study was to compare the predictive performance and user convenience of the PRE-DELIRIC and E-PRE-DELRIC models. Second, we sought to determine the value of the use of both models in a two-stage calculation of patients’ risk of delirium in the ICU (i.e. the E-PRE-DELIRIC model immediately after ICU admission with an updated delirium risk score after 24 h of ICU admission using the PRE-DELIRIC model) to see if we could expand on current models, since it is well-known that dynamic variables assessed over time as opposed to variables assessed at admission only tend to perform better in prediction models.

## Methods

### Design and study population

The “Delirium prediction in the intensive care unit: comparison of two delirium prediction models” (DECISION) study was a multinational prospective cohort study conducted in 11 ICUs from seven different countries (Australia, Belgium, Canada, Denmark, Portugal, USA, and the Netherlands). Each study site had a well-established delirium screening protocol and similar delirium treatment practices. All consecutive, critically ill adults admitted to the ICU were enrolled. Patients were excluded if they had delirium at the time of ICU admission, were discharged from the ICU within 6 h, or were unable to be reliably assessed for delirium (e.g. sustained coma, inability to understand the predominant language spoken in the ICU, severe cognitive dysfunction, receptive aphasia, or serious auditory or visual disorders) [9–11]. Each institution enrolled patients for up to three months or until data on 300 patients were collected.

### Data collection

Data were collected over the first 14 days of the ICU stay. Data for each delirium predictor (nine predictors for the E-PRE-DELIRIC model and ten for the PRE-DELIRIC model) were collected in consecutive patients immediately after ICU admission (E-PRE-DELIRIC) [11] and within 24 h of ICU admission (PRE-DELIRIC) [9, 10] and entered into a validated web-based, data management system, Castor [15]. Severity of illness was estimated at ICU admission using the Acute Physiology and Chronic Health Evaluation (APACHE) II score [16] and daily using the Sequential Organ Failure Assessment (SOFA) score [17].

The presence of delirium in the ICU was evaluated at least every 12 h by the trained bedside nurse using either the Confusion Assessment Method for the Intensive Care Unit (CAM-ICU) [18] or the Intensive Care Delirium Screening Checklist (ICDSC) [19]. Development of delirium in the ICU was defined as at least one positive assessment of delirium using the CAM-ICU or ICDSC. Patients were also deemed to have delirium whenever they were administered haloperidol or an atypical antipsychotic drug for treatment of delirium, to prevent false negative delirium screenings. To eliminate bias, nurses were kept unaware of the fact that their delirium assessments were used for a study [20].

Level of sedation (using either the Richmond Agitation-Sedation Scale (RASS) or the Riker Sedation-Agitation Scale (SAS) [21, 22], and current intravenous (IV) sedative therapy was documented at the time each delirium assessment was completed. Delirium was preferentially evaluated when patients were maximally awake (e.g. after a spontaneous awakening trial). When coma was present (i.e., RASS = − 4 or − 5 or Riker-SAS = 1 or 2) patients were designated as unable to be assessed for delirium.

To help ensure that the nurse delirium assessments were of high quality [23], a trained investigator (or research nurse) independently and sequentially evaluated patients for the presence of delirium using the same tool as the bedside nurse (i.e., CAM-ICU or ICDSC) during one daytime shift each month and nurse-expert inter-rater reliability (IRR) was calculated. A delirium assessment compliance rate (i.e. delirium assessments documented/delirium assessments that should have been completed) was calculated for one day monthly in each ICU. If Cohen’s kappa for the paired delirium assessments was ≥ 0.80 and delirium screening compliance was ≥ 80% then the delirium assessment was considered to be reliable in that ICU. Prior to the study it was determined that centres would be described separately if they did not meet these two reliability criteria and if they had outcomes that significantly affected the performance of the (E)-PRE-DELIRIC model in the primary analysis.

### Evaluation of the user convenience of the delirium prediction model

To estimate delirium model user convenience, the preferences of ICU physicians regarding the two delirium prediction models was determined by electronically administering a short, optional, and anonymous web-based survey (comprising four 5-point, Likert-scale questions) to all physicians working in each study ICU (Additional file 1). A completed questionnaire indicated that a physician provided consent for their data to be used.

### Statistical analysis

For each delirium prediction model at least 200 events, i.e. positive delirium assessments, were needed [24]. With an anticipated delirium incidence conservatively set at 20%, we aimed to enrol 2000 patients in total (400/0.20 = 2000 patients).

The discriminative power of both models was assessed using the area under the receiver-operating characteristic curve (AUROC) [20]. The database was divided into groups based on the quartiles of the predicted probabilities for delirium development: very low (0.00–0.10), low (0.10–0.20), moderate (0.20–0.30), and high risk of delirium (≥ 0.30).

Sensitivity, specificity, and likelihood ratios were calculated for these four groups. Calibration was assessed graphically by plotting the observed outcome frequencies against the mean predicted outcome probabilities or risks, within subgroups of patients that were ranked by increasing estimated probability [25]. The predictive performance of both models was compared using the Hanley and McNeil method [26]. It is estimated that approximately a third of patients will develop delirium in the ICU [3]. We therefore rated patients with a predicted probability of delirium <0.30 as low-risk patients and with probability ≥0.30 as high-risk patients. The additional value of a two-stage calculation in low-risk patients was determined using the E-PRE-DELIRIC model to calculate a patient’s risk of delirium immediately after ICU admission. Subsequently we used data from the first 24 h in ICU to update the prediction using the PRE-DELIRIC model to determine how many patients with a probability of delirium <0.30 predicted using the E-PRE-DELIRIC model would subsequently be labelled at high risk of developing delirium using the PRE-DELIRIC score. Both risk calculations were compared to the patients’ delirium outcome.

The questionnaires for ICU physicians were analyzed using the Wilcoxon signed ranks test for non-parametric statistical testing of two dependent samples. Statistical significance was defined as *p* < 0.05 and the null hypotheses were tested against two-sided alternatives. Data were analysed using SPSS® Statistics version 22 and R statistics R3.2.4 [27].

## Results

**Fig. 1:**
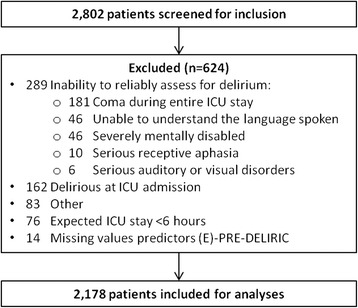
Study flowchart. E-PRE-DELIRIC, early prediction model for delirium in ICU patients

A total of 2802 patients were screened for inclusion; 2178 patients (78%) were included. Among the 624 patients excluded, inability to reliably assess for delirium (46.3% (289/624)) and delirium at the time of ICU admission (25.9% (162/624)) were the most common reasons for exclusion (see study flowchart, Fig. 1). Among the 83 patients that were excluded for other reasons the most common reasons were severe neurological injury and confidential files. Patients were 62.1 ± 15.2 years old, 60.8% male, and had a baseline APACHE-II score of 17.4 ± 7.1. During their ICU stay 21.4% of patients (467/2178) developed delirium. Patient characteristics are presented in Table 1. For patient and hospital characteristics per participating ICU see Additional file 2: Table S1.

**Table 1:** Patient characteristics

Variable	Total cohort (*N* = 2178)
Age in years, mean (SD)	62.1 (15.2)
Male, *N* (%)	1324 (60.8)
Admission category, *N* (%)	
-Surgery	1079 (49.5)
-Medical	859 (39.3)
-Trauma	86 (4.0)
-Neurology/neurosurgery	157 (7.2)
Urgent admission, *N* (%)	1345 (61.8)
Use of sedatives during ICU stay for ≥ 1 day, *N* (%)	992 (45.5)
Comatose during ICU stay for ≥ 1 day, *N* (%)	873 (40.1)
E-PRE-DELIRIC score, median (Q1–Q3, min/max)	16.7 (9–32, 2/99)
PRE-DELIRIC score, median (Q1–Q3, min/max)	18.4 (12–30, 3/98)
SOFA score, median (Q1–Q3, min/max)	4.5 (3.0–6.6, 1/20)
APACHE-II score, mean (SD)	17.4 (7.1)
Delirium, *N* (%)	467 (21.4)
-Positive delirium assessment	431 (19.7)
-Positive based on medication for delirium treatment	35 (1.7)
LOS-ICU in days, median (Q1–Q3, min/max)	3.0 (2–6, 1/96)

Sedatives = IV sedative therapy. Level of sedation was assessed using either the Richmond Agitation-Sedation Scale (RASS) or the Riker Sedation-Agitation Scale (SAS) [21, 22]. Coma = RASS = − 4 or − 5 or Riker-SAS = 1 or 2

*SOFA* Sequential Organ Failure Assessment score [17], *APACHE-II* the Acute Physiology and Chronic Health Evaluation II [16], *LOS-ICU* length of stay in the intensive care unit, *PRE-DELIRIC* prediction model for delirium in ICU patients, *E-PRE*-*DELIRIC* early prediction model for delirium in ICU patients

### Model performance

**Fig. 2:**
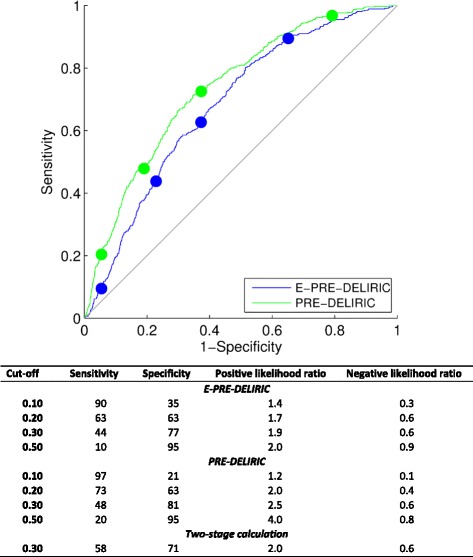
AUROC for the early prediction model for delirium in ICU patients (E-PRE-DELIRIC) and the prediction model for delirium in ICU patients (PRE-DELIRIC)

**Fig. 3:**
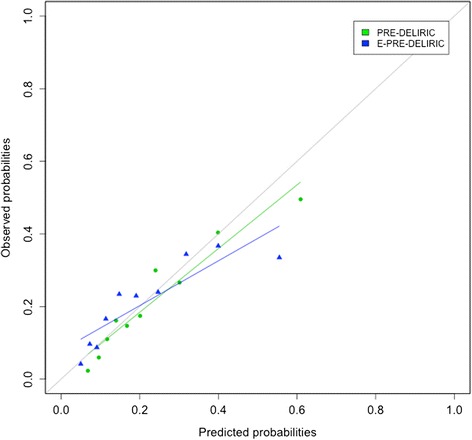
Calibration plot for the early prediction model for delirium in ICU patients (E-PRE-DELIRIC) and the prediction model for delirium in ICU patients (PRE-DELIRIC)

The AUROC of the PRE-DELIRIC model (0.74 (95% CI 0.71–0.76)) was significantly greater than that for the E-PRE-DELIRIC model (0.68 (95% CI 0.66–0.71)) (*z* score of − 2.73 (*p* < 0.01)) (Fig. 2). Both models were well-calibrated (Fig. 3).

At a cutoff of 0.214, the incidence of delirium was 21.4% in the total sample, and the sensitivity and specificity were 60 and 65%, respectively, for the E-PRE-DELIRIC model and 69 and 66% for the PRE-DELIRIC model.

### Two-stage calculation

A total of 1586 patients had a predicted probability of delirium <0.30 with the E-PRE-DELIRIC model and therefore were not deemed to be at high risk of delirium based on the cutoff of 0.30. However, 262 of these patients eventually did experience delirium during their ICU stay, despite an initial low predicted risk of delirium calculated using the E-PRE-DELIRIC model. Using data from the first 24 h in the ICU, the PRE-DELIRIC model identified 64 of these 262 patients (24%) to be at high risk of delirium (i.e. predicted probability of delirium ≥0.30). The two-stage calculation improved the sensitivity of the prediction by 14% compared to the E-PRE-DELIRIC model alone and by 10% compared to the PRE-DELIRIC model alone (Fig. 2).

### User convenience

In total, 68 (41%) ICU physicians completed the user convenience questionnaire. Of the ICU physicians who participated in this study 52 (76%) were intensivists, 11 (16%) were intensivist trainees, 4 (6%) were specialists other than intensivists, and 1 (2%) was a non-specialist physician. There were 56 ICU physicians (82%) with no prior experience of using a delirium prediction model; only 1 physician had experience of using the E-PRE-DELIRIC model and 11 of using the PRE-DELIRIC model: none of the physicians used a prediction model regularly, although 6 physicians (9%) stated that a prediction model had been implemented in the ICU where they work. Physicians perceived that the PRE-DELIRIC model (versus the E-PRE-DELIRIC model) took more “time and effort to collect data” (*p* < 0.05) and was a greater “burden for the physician to collect the model data” (*p* < 0.01). In contrast, for the E-PRE-DELIRIC model, physicians perceived that “the predictors were more available” (*p* < 0.05) and that they were more likely “to use this model (vs. the PRE-DELIRIC model) in daily practice” (*p* < 0.05). The “clearness of the definitions” and the “reliability of the outcome” of the E-PRE-DELIRIC and PRE-DELIRIC models were perceived to be similar (Table 2 and Additional file 3: Table S2).

**Table 2:** Outcome user convenience questionnaire

Question	Negative ranks^ (*N*)	Ties^ (*N*)	Positive ranks^ (*N*)	Significance*
Time and effort needed to collect data to calculate a patient’s risk	4	45	16	*p* < 0.05
Burden for the physician to collect data about the predictors to calculate a patient’s risk	3	42	20	*p* < 0.01
Availability of predictors	11	50	4	*p* < 0.05
Clearness of the definitions of the predictors	5	56	4	*p* = 1.00
Reliability of the outcome (predicted risk) of the prediction model	2	62	1	*p* > 0.5
Are you going to use the delirium prediction model in daily practice?	9	55	1	*p* < 0.05

^Prediction model for delirium in ICU patients (PRE-DELIRIC) compared to the early prediction model for delirium in ICU patients (E-PRE-DELIRIC): negative ranks indicate the number of ICU physicians who scored PRE-DELIRIC lower; ties indicate no difference; positive ranks indicate the number of ICU physicians who scored PRE-DELIRIC higher

*Null hypotheses were tested against two-sided alternatives

### Quality check of the delirium assessment

The overall quality of delirium assessment and screening compliance were strong (see Additional file 4: Table S3 for all IRR and compliance rates for each participating ICU). When the ICUs that did not fully meet all delirium assessment reliability criteria were removed from the analysis, as was determined a priori, neither the performance of the E-PRE-DELIRIC model nor the PRE-DELIRIC model was significantly affected. Consequently all centres were included in the primary analysis.

## Discussion

This large, multinational prospective cohort study provides insight into the comparative performance of two available ICU delirium prediction models (i.e. the E-PRE-DELIRIC model that estimates the risk of delirium at the time of ICU admission and the PRE-DELIRIC model used to estimate the risk of delirium 24 h later) [9–11]. Both models had a moderate-to-good statistical performance. Although the predictive accuracy of the E-PRE-DELIRIC model was somewhat lower, its user convenience appeared to be better compared to the PRE-DELIRIC model. To allow for optimal implementation of a delirium prediction model in daily practice, involvement and the opinion on user convenience of the target group, i.e. the ICU physicians, is very important [28, 29]. Based on these results, the E-PRE-DELIRIC model is likely the model that can be implemented most successfully in daily ICU practice. Moreover, our analysis indicates that when the E-PRE-DELIRIC model predicts a low risk of delirium, an additional calculation using the PRE-DELIRIC model after 24 h in the ICU increases the model’s sensitivity to detect patients that will develop delirium who are incorrectly identified as low-risk patients. Thus, this method will prevent deprivation of delirium-preventive measures in patients with a false negative rating (i.e. the patients with predicted probability <0.30 who develop delirium during ICU admission).

The routine use of delirium preventive measures in the ICU is widely endorsed given the high prevalence of delirium, its deleterious effects on patient outcome [30, 31], and the high costs related to these effects [32]. The routine use of a delirium prediction model may facilitate early recognition of those patients at greatest risk of delirium who may benefit the most from delirium preventive measures [32]. Importantly, when resources are limited, the use of delirium risk stratification to target high-risk patients makes wider implementation of multicomponent non-pharmacological interventions aimed at preventing delirium more feasible [32]. Preventive measures should be initiated as soon as possible after ICU admission; therefore an early ICU delirium prediction model is preferred.

The performance of a prediction model outside the development sample determines its generalisability in clinical practice [33]. External validation of many other clinical prediction models is lacking [34]. Of interest, both the E-PRE-DELIRIC and recalibrated PRE-DELIRIC models are validated externally and had moderate-to-good statistical performance in independent data sets, allowing for generalisation to non-study ICUs around the world [[35], Wassenaar et al. 2017]. “External validation of two models to predict delirium in intensive care unit patients. Unpublished data.” The delirium incidence, estimated based on new positive delirium assessments after ICU admission, is important for the performance and thus the generalisation of both delirium prediction models. One might argue that the delirium incidence in our study cohort was relatively low. However, multiple previous studies have reported comparable delirium incidence rates [36–38].

Our study has important strengths. The use of a cohort design without strict eligibility criteria helped boost its generalisability [14], and its prospective nature allowed us to carefully measure and document the predictors and outcomes, thereby improving its applicability and reproducibility in non-study ICUs [14, 20, 39]. The large number of patients enrolled, their mixed characteristics, and the multinational character of our study allows the results to be applied in the vast majority of ICUs in the developed world. Of note, when generalising to high-intensity ICUs it should be taken into account that their patient group will probably have more severe illness in comparison with our study cohort. For future research it might be of interest to study the performance of both models in patients with APACHE and SOFA scores in the higher ranges. The proportion of ICU physicians responding was better than response rates shown in other physician surveys [40]. No imputation techniques were used to handle missing data, as we wanted to determine the clinical performance of both delirium prediction models and the use of a prediction model in daily clinical practice does not allow for imputation. Our efforts to provide clear definitions and instruction manuals to all study sites resulted in the exclusion of only fourteen patients due to missing values for the predictors.

Several limitations are also present. It might be possible that the two delirium prediction models evaluated might need to be updated in the future as new risk factors for delirium in the ICU may emerge. Of course, this also offers an opportunity to further improve the discriminative performance of each model, which in particular could benefit the E-PRE-DELIRIC. For an update, referred to as model revision, one needs to have insight into new risk factors for delirium, both available at ICU admission or within 24 h of ICU admission. Subsequently, a new prediction study is needed to determine which of the new risk factors improves the performance of the models and should be used for model revision [41]. It is important to realize that when a model is used to predict a patient’s risk of an event, it should always be considered an approximation no matter how strong the documented predictive accuracy. This is particularly important in the case of medical decision making. Two well-validated delirium screening-instruments (i.e. the CAM-ICU and the ICDSC) were used in this study. Naturally, the sensitivity and specificity of each instrument differs [42]. Realizing that the sensitivity of either screening tool is not 100%, we also defined delirium to be present when haloperidol or an atypical antipsychotic was administered for the treatment of delirium. While each ICU had similar delirium treatment protocols, we cannot exclude that antipsychotic therapy may have been initiated in patients who did not have delirium.

It is shown that ICU clinicians’ predictions are less accurate than those of an ICU delirium prediction model [9]. We believe that routine prognostic delirium evaluation in the ICU is important in the clinical setting to identify those patients who may benefit the most from early preventive measures and in the research setting to ensure that delirium risk is well-characterized and stratified in controlled studies. We want to emphasize that the predicted risk score is an estimation of the chance of developing delirium during ICU admission that may facilitate early clinical decisions on delirium prevention and personalized care to ICU patients and their family members. However, the rationing of critical care resources should not be based on the predicted risk score for delirium over the first 24 h of ICU admission alone as the predicted risk score does not take into account changes in the health status of ICU patients. For future controlled studies on the effect of both non-pharmacological and pharmacological interventions on ICU delirium, we suggest stratifying patients based on their risk of delirium and restricting delirium prevention to those patients at high risk of delirium.

To achieve the best predictive performance and user convenience currently possible, we suggest a two-stage calculation using the E-PRE-DELIRIC model in all patients admitted to the ICU to predict patients’ risk of delirium immediately after ICU admission and to update the risk scores of the patients at low risk of delirium after 24 h using the PRE-DELIRIC model. This way, the chance of missing a patient that will develop delirium during ICU admission is further attenuated. Still, a substantial minority of the patients that develop delirium during ICU admission will score a low predicted risk for delirium using both delirium prediction models in a two-stage calculation. Of interest, in this study cohort the delirium incidence was 21%. Based on the fact that the sensitivity and specificity of a prediction model are most optimal at the cutoff of the incidence level of the outcome of interest, in this case delirium, we expect that the sensitivity in a population with around 30% incidence of delirium will be better compared to the sensitivity shown in our study. Of importance, the acceptability of the suggested two-stage calculation by ICU physicians is not yet assessed. Future research should focus on the usefulness of both delirium prediction models in clinical practice and their impact on clinical outcomes, since this is the only way to determine whether their use improves usual care [41]. In addition, such an impact analysis also provides the opportunity to study the acceptance of the models in daily practice [41], in which it is interesting to also take the experiences of ICU patients and their families into account.

## Conclusions

This study shows that statistically both ICU delirium prediction models have moderate-to-good performance. Although the predictive accuracy of the PRE-DELIRIC is greater, the E-PRE-DELIRIC model scores significantly better on user convenience. Moreover, the PRE-DELIRIC model needs data obtained during a period of 24 h, while the E-PRE-DELIRIC can be obtained at ICU admission, allowing direct preventive measures and stratified randomization in studies at the time of ICU admission. In patients who appear to be at low risk of delirium at ICU admission, it is of additional advantage to update their predicted risk scores using the PRE-DELIRIC model after 24 h in the ICU.

## Additional files

Additional file 1: Physician questionnaire DECISION study. (DOCX 27 kb)

Additional file 2: **Table S1.** Patient and hospital characteristics. (DOCX 22 kb)

Additional file 3: **Table S2.** Outcome user convenience questionnaire for ICU physicians. (DOCX 19 kb)

Additional file 4: **Table S3.** Quality check delirium assessment. (DOCX 18 kb)

## Abbreviations

AUROC: Area under the receiver operating characteristic curve
CAM-ICU: Confusion Assessment Method for the ICU
E-PRE-DELIRIC model: Early prediction model for delirium in ICU patients
ICDSC: Intensive Care Delirium Screening Checklist
ICU: Intensive care unit
IRR: Inter-rater reliability
PRE-DELIRIC model: Prediction model for delirium in ICU patients

## References

[1] APA (2013). Diagnostic and statistical manual of mental disorders, fifth edition.

[2] Milbrandt EB, Deppen S, Harrison PL (2004). Costs associated with delirium in mechanically ventilated patients. Crit Care Med.

[3] Salluh JI, Wang H, Schneider EB (2015). Outcome of delirium in critically ill patients: systematic review and meta-analysis. BMJ.

[4] Mistraletti G, Pelosi P, Mantovani ES, Berardino M, Gregoretti C (2012). Delirium: clinical approach and prevention. Best Pract Res Clin Anaesthesiol.

[5] Altman DG, Royston P (2000). What do we mean by validating a prognostic model?. Stat Med.

[6] Giannini A, Garrouste-Orgeas M, Latour JM (2014). What’s new in ICU visiting policies: can we continue to keep the doors closed?. Intensive Care Med.

[7] Ely EW (2017). The ABCDEF bundle: science and philosophy of how ICU liberation serves patients and families. Crit Care Med.

[8] Morandi A, Piva S, Ely EW (2017). Worldwide survey of the “Assessing Pain, Both Spontaneous Awakening and Breathing Trials, Choice of Drugs, Delirium Monitoring/Management, Early Exercise/Mobility, and Family Empowerment” (ABCDEF) bundle. Crit Care Med.

[9] van den Boogaard M, Pickkers P, Slooter AJ (2012). Development and validation of PRE-DELIRIC (PREdiction of DELIRium in ICu patients) delirium prediction model for intensive care patients: observational multicentre study. BMJ.

[10] van den Boogaard M, Schoonhoven L, Maseda E (2014). Recalibration of the delirium prediction model for ICU patients (PRE-DELIRIC): a multinational observational study. Intensive Care Med.

[11] Wassenaar A, van den Boogaard M, van Achterberg T (2015). Multinational development and validation of an early prediction model for delirium in ICU patients. Intensive Care Med.

[12] Ely EW, Shintani A, Truman B (2004). Delirium as a predictor of mortality in mechanically ventilated patients in the intensive care unit. JAMA.

[13] Serafim RB, Dutra MF, Saddy F (2012). Delirium in postoperative nonventilated intensive care patients: risk factors and outcomes. Ann Intensive Care.

[14] Moons KG, Royston P, Vergouwe Y, Grobbee DE, Altman DG (2009). Prognosis and prognostic research: what, why, and how?. BMJ.

[15] Castor Electronic Data Capture. Amsterdam, the Netherlands: Ciwit BV; 2017.

[16] Knaus WA, Draper EA, Wagner DP, Zimmerman JE (1985). APACHE II: a severity of disease classification system. Crit Care Med.

[17] Vincent JL, Moreno R, Takala J (1996). The SOFA (Sepsis-related Organ Failure Assessment) score to describe organ dysfunction/failure. On behalf of the Working Group on Sepsis-Related Problems of the European Society of Intensive Care Medicine. Intensive Care Med.

[18] Ely EW, Inouye SK, Bernard GR (2001). Delirium in mechanically ventilated patients: validity and reliability of the confusion assessment method for the intensive care unit (CAM-ICU). JAMA.

[19] Bergeron N, Dubois MJ, Dumont M, Dial S, Skrobik Y (2001). Intensive Care Delirium Screening Checklist: evaluation of a new screening tool. Intensive Care Med.

[20] Moons KG, Kengne AP, Woodward M (2012). Risk prediction models: I. Development, internal validation, and assessing the incremental value of a new (bio)marker. Heart.

[21] Riker RR, Picard JT, Fraser GL (1999). Prospective evaluation of the Sedation-Agitation Scale for adult critically ill patients. Crit Care Med.

[22] Sessler CN, Gosnell MS, Grap MJ (2002). The Richmond Agitation-Sedation Scale: validity and reliability in adult intensive care unit patients. Am J Respir Crit Care Med.

[23] van Eijk MM, van den Boogaard M, van Marum RJ (2011). Routine use of the confusion assessment method for the intensive care unit: a multicenter study. Am J Respir Crit Care Med.

[24] Collins GS, Ogundimu EO, Altman DG (2016). Sample size considerations for the external validation of a multivariable prognostic model: a resampling study. Stat Med.

[25] Steyerberg EW (2009). Clinical prediction models: a practical approach to development, validation, and updating.

[26] Hanley JA, McNeil BJ (1983). A method of comparing the areas under receiver operating characteristic curves derived from the same cases. Radiology.

[27] R Core Team. R: A language and environment for statistical computing. Vienna: 2017. http://www.R-project.org/

[28] Grol R, Wensing M (2004). What drives change? Barriers to and incentives for achieving evidence-based practice. Med J Aust.

[29] Grol R, Wensing M: Implementation; Effective improvement of patient care. (In Dutch: Implementatie; Effectieve Verbeteringen Van Patiëntenzorg. Amsterdam: Bohn Stafleu en van Loghum; 2016.

[30] Barr J, Fraser GL, Puntillo K (2013). Clinical practice guidelines for the management of pain, agitation, and delirium in adult patients in the intensive care unit. Crit Care Med.

[31] NICE (2010). DELIRIUM: diagnosis, prevention and management.

[32] Siddiqi N (2016). Predicting delirium: time to use delirium risk scores in routine practice?. Age Ageing.

[33] Altman DG, Vergouwe Y, Royston P, Moons KG (2009). Prognosis and prognostic research: validating a prognostic model. BMJ.

[34] Siontis GC, Tzoulaki I, Castaldi PJ, Ioannidis JP (2015). External validation of new risk prediction models is infrequent and reveals worse prognostic discrimination. J Clin Epidemiol.

[35] Lee A, Mu JL, Joynt GM (2017). Risk prediction models for delirium in the intensive care unit after cardiac surgery: a systematic review and independent external validation. Br J Anaesth.

[36] van den Boogaard M, Schoonhoven L, van der Hoeven JG, van Achterberg T, Pickkers P (2012). Incidence and short-term consequences of delirium in critically ill patients: a prospective observational cohort study. Int J Nurs Stud.

[37] Peterson JF, Pun BT, Dittus RS (2006). Delirium and its motoric subtypes: a study of 614 critically ill patients. J Am Geriatr Soc.

[38] Woien H, Balsliemke S, Stubhaug A (2013). The incidence of delirium in Norwegian intensive care units; deep sedation makes assessment difficult. Acta Anaesthesiol Scand.

[39] Simon R, Altman DG (1994). Statistical aspects of prognostic factor studies in oncology. Br J Cancer.

[40] Dykema J, Jones NR, Piche T, Stevenson J (2013). Surveying clinicians by web: current issues in design and administration. Eval Health Prof.

[41] Moons KG, Altman DG, Vergouwe Y, Royston P (2009). Prognosis and prognostic research: application and impact of prognostic models in clinical practice. BMJ.

[42] Gusmao-Flores D, Salluh JI, Chalhub RA, Quarantini LC (2012). The Confusion Assessment Method for the Intensive Care Unit (CAM-ICU) and Intensive Care Delirium Screening Checklist (ICDSC) for the diagnosis of delirium: a systematic review and meta-analysis of clinical studies. Crit Care.

