# Supplementary material for: Delirium prediction in the intensive care unit: comparison of two delirium prediction models
Source: Crit Care. 2018 May 5;22:114. doi: 10.1186/s13054-018-2037-6 (PMC5935943; doi:10.1186/s13054-018-2037-6)
Supplement: Supplementary file 1 — Physician questionnaire DECISION study. (DOCX 27 kb) [file 13054_2018_2037_MOESM1_ESM.docx]

**Additional file 1:** Physician Questionnaire DECISION study

Hospital: ……………

Date: …………… ……………………………………

By means of this questionnaire we want to determine your opinion about the *user convenience* of both delirium prediction models (E-PRE-DELIRIC and PRE-DELIRIC).

| **E-PRE-DELIRIC:**  Delirium prediction immediately after  ICU admission | **PRE-DELIRIC:**  Delirium prediction within 24 hours after  ICU admission |
| --- | --- |
| **Predictors*:** | |
| Age (years) | Age (years) |
| Blood urea nitrogen (BUN) (mmol/L) | Blood urea nitrogen (BUN) (mmol/L) |
| Admission category | Admission category |
| Urgent admission | Urgent admission |
| *History of alcohol abuse* | *Coma* |
| *History of cognitive impairment* | *Infection* |
| *Mean arterial blood pressure* | *Morphine use* |
| *Use of corticosteroids* | *Sedative use* |
| *Respiratory failure* | *APACHE-II score (per point)* |
|  | *Metabolic acidosis (pH <7.35, with bicarbonate <24 mmol/L)* |

**Italic fond in table: predictors that differ from each other per prediction model*

1. *What is your profession in ICU?*

❑ Intensivist

❑ Intensivist trainee

❑ Specialist, other than intensivist

❑ Physician without specialization

1. *Have you previously worked with a delirium prediction model?*

❑ no, no prior experience with a delirium prediction model

❑ yes, in this unit (E-PRE-DELIRIC)

❑ yes, in this unit (PRE-DELIRIC)

❑ yes, elsewhere (E-PRE-DELIRIC)

❑ yes, elsewhere (PRE-DELIRIC)

If yes

❑ implemented in daily practice

❑ use on occasion

1. *What do you think of the user convenience of both delirium prediction models, in terms of:*

(Please place for both delirium prediction models an X in the box behind the answer of your choice)

|  | **Possible**  **answers** | **E-PRE-DELIRIC** | **PRE-DELIRIC** |
| --- | --- | --- | --- |
| Time and effort needed to collect data to calculate a patient’s risk | Very low |  |  |
|  | Low |  |  |
|  | Neutral |  |  |
|  | High |  |  |
|  | Very high |  |  |
|  | | | |
| Burden for the physician to collect data about the predictors to calculate a patient’s risk | Very low |  |  |
|  | Low |  |  |
|  | Neutral |  |  |
|  | High |  |  |
|  | Very high |  |  |
|  | | | |
| Availability of predictors | Never available |  |  |
|  | Unavailable |  |  |
|  | Neutral |  |  |
|  | Available |  |  |
|  | Always available |  |  |
|  | | | |
| Clearness of the definitions of the predictors | Very vague |  |  |
|  | Vague |  |  |
|  | Neutral |  |  |
|  | Clear |  |  |
|  | Very clear |  |  |
|  | | | |
| Reliability of the outcome (predicted risk) of the prediction model | Very unreliable |  |  |
|  | Unreliable |  |  |
|  | Neutral |  |  |
|  | Reliable |  |  |
|  | Very reliable |  |  |
|  | | | |
| Are you going to use the delirium prediction model in daily practice | Not |  |  |
|  | Probably not |  |  |
|  | Neutral |  |  |
|  | Probably |  |  |
|  | Sure |  |  |
|  | Depends on this study |  |  |
|  | | | |

1. *Do you have other information you like to add concerning the delirium prediction models?*

Thank you for your response!
