# Supplementary material for: Delirium prediction in the intensive care unit: comparison of two delirium prediction models
Source: Crit Care. 2018 May 5;22:114. doi: 10.1186/s13054-018-2037-6 (PMC5935943; doi:10.1186/s13054-018-2037-6)
Supplement: Supplementary file 2 — Table S1. Patient and hospital characteristics. (DOCX 22 kb) [file 13054_2018_2037_MOESM2_ESM.docx]

**Additional file 2:Table S1** Patient and hospital characteristics

| **Variable** | **Total cohort**  **(N=2,178)** | **University Medical Centre Utrecht (UMCU)**  **(N=292)** | **Jeroen Bosch Ziekenhuis (JBZ)**  **(N=146)** | **Antwerp University Hospital (AUH)**  **(N=288)** | **The Canberra Hospital (TCH)**  **(N=299)** | **Medisch Spectrum Twente (MST)**  **(N=64)** | **Hospital Espírito Santo (HES)**  **(N=48)** | **Erasmus Medical Center (EMC)**  **(N=222)** | **Tufts Medical Center (TMC)**  **(N=274)** | **Radboud university medical center (RUMC)**  **(N=298)** | **Rigs**  **hospitalet**  **(RHL)**  **(N=139)** | **Mt Sinai Hospital/U of Toronto (SHT)**  **(N=108)** |
| --- | --- | --- | --- | --- | --- | --- | --- | --- | --- | --- | --- | --- |
| Age in years, Mean (SD) | 62.1 (15.2) | 62.8 (14.2) | 63.0 (15.2) | 62.6 (14.6) | 63.6 (16.5) | 61.5 (12.4) | 69.4 (13.0) | 56.1 (15.7) | 63.1 (15.2) | 63.1 (14.0) | 62.2 (14.6) | 57.4 (17.5) |
| Male, N (%) | 1324 (60.8) | 193 (66.1) | 78 (53.4) | 171 (59.4) | 172 (57.5) | 44 (68.8) | 43 (89.6) | 141 (63.5) | 169 (61.7) | 189 (63.4) | 92 (66.2) | 43 (39.8) |
| Admission category, N (%)  Surgery  Medical  Trauma  Neurology/neurosurgery | 1079 (49.5)  859 (39.3)  86 (4.0)  157 (7.2) | 191 (65.4)  58 (19.9)  10 (3.4)  33 (11.3) | 41 (28.1)  100 (68.5)  3 (2.0)  2 (1.4) | 159 (55.2)  112 (38.9)  17 (5.9)  - | 129 (43.1)  134 (44.8)  19 (6.4)  17(5.7) | 17 (26.6)  42 (65.6)  2 (3.1)  3 (4.7) | 12 (25.0)  33 (68.8)  2 (4.1)  1 (2.1) | 89 (40.1)  66 (29.7)  14 (6.3)  53 (23.9) | 101 (36.9)  164 (59.8)  1 (0.4)  8 (2.9) | 208 (69.8)  56 (18.8)  11 (3.7)  23 (7.7) | 72 (51.8)  43 (30.9)  7 (5.0)  17 (12.2) | 60 (55.6)  48 (44.4)  -  - |
| Urgent admission, N (%) | 1345 (61.8) | 108 (37.0) | 126 (86.3) | 173 (60.1) | 216 (72.2) | 57 (89.1) | 48 (100) | 112 (50.5) | 241 (88.0) | 104 (34.9) | 88 (63.3) | 72 (66.7) |
| ≥1 day use of sedatives during ICU stay, N (%) | 992 (45.5) | 72 (24.7) | 74 (50.7) | 158 (54.9) | 109 (36.5) | 56 (87.5) | 40 (83.3) | 142 (64.0) | 128 (46.7) | 105 (35.2) | 67 (48.2) | 41 (38.0) |
| ≥1 day comatose during ICU stay, N (%) | 873 (40.1) | 60 (20.5) | 46 (31.5) | 155 (53.8) | 102 (34.1) | 46 (31.5) | 39 (81.3) | 73 (32.9) | 91 (33.2) | 158 (53.0) | 77 (55.4) | 24 (22.2) |
| E-PRE-DELIRIC score, Median (Q1-Q3, min/max) | 16.7  (9-32, 2/99) | 10.2  (7-23,  2/68) | 25.1  (15-37,  4/100) | 16.4  (9-33,  2/75) | 19.0  (11-31,  3/65) | 30.4  (20-40,  5/56) | 48.7  (37-62,  22/82) | 12.7  (7-22,  3/58) | 18.8  (13-28,  3/71) | 10.4  (8-18,  2/76) | 29.8  (19-48,  10/100) | 10.7  (6-21, 3/96) |
| PRE-DELIRIC score,  Median (Q1-Q3, min/max) | 18.4  (12-30, 3/98) | 18.0  (11-28,  4/84) | 19.4  (13-31,  5/68) | 17.6  (11-41,  4/94) | 16.7  (11-27,  4/78) | 29.7  (20-40,  7/51) | 43.4  (33-58,  12/83) | 21.0  (14-29,  5/80) | 18.5  (13-25,  5/59) | 14.0  (11-23,  3/78) | 39.6  (27-52,  7/99) | 12.3  (9-21, 4/96) |
| SOFA,  Median (Q1-Q3, min/max) | 4.5  (3.0-6.6,  1/20) | 4.0  (3.0-6.0,  1/16) | N.A. | 6.0  (4.0-8.0,  1/19) | 4.0  (3.0-5.8,  1/13) | 6.5  (3.0-6.5,  3/10) | 6.4  (5.3-8.3,  2/14) | 5.9  (4.0-7.9,  1/20) | 3.4  (2.0-5.1,  1/15) | N.A. | N.A. | 3.0  (1.6-5.0,  1/15) |
| APACHE-II score, Mean (SD) | 17.4 (7.1) | 17.3 (6.0) | 19.2 (7.7) | 17.2 (6.9) | 16.2 (6.7) | 18.7 (6.0) | 24.3 (8.0) | 18.6 (6.6) | 15.0 (6.2) | 16.1 (5.5) | 23.9 (7.7) | 14.8 (8.7) |
| Delirium, N (%) | 467 (21.4) | 58 (19.9) | 36 (24.7) | 64 (22.2) | 37 (12.4) | 24 (37.5) | 5 (10.4) | 59 (26.6) | 66 (24.1) | 71 (23.8) | 32 (23.0) | 14 (13.0) |
| LOS-ICU in days,  Median (Q1-Q3, min/max) | 3 (2-6, 1/96) | 2 (2-4, 1/45) | 3 (2-6, 1/36) | 3 (2-6, 1/85) | 3 (2-5, 1/96) | 7 (3-17,  1/81) | 6 (3-12,  2/19) | 3 (2-7, 1/57) | 3 (2-6, 1/14) | 2 (2-3, 1/71) | 4 (2-8, 2/38) | 3 (2-5, 1/49) |
| Delirium assessment:  Tool  Number of assessments/day  Implementation | NA | CAM-ICU  2/day  2010 | CAM-ICU  3/day  2009 | ICDSC  3/day  2015-2016 | CAM-ICU  3/day  2012 | CAM-ICU  3/day  2013 | CAM-ICU  2/day  2015 | ICDSC  3/day  2012-2014 | ICDSC  2/day  2005 | CAM-ICU  3/day  2008 | CAM-ICU  2-3/day  2012-2015 | ICDSC  2/day  2010 |
| Number of beds  Yearly admission rate | NA | 30  2000  Mixed ICU | 16 beds  850  Mixed ICU | 45 beds  2800  Mixed ICU | 31 beds  2000  Mixed ICU | 18  900  Mixed ICU | 5-6 beds  200  Medical ICU | 30 beds  1200-1300  Mixed ICU | 30 beds  3300  Mixed ICU | 34 beds  2500  Mixed ICU | 40 beds  2600  Mixed ICU | 16 beds  800-850  Mixed ICU |

Sedatives = IV sedative therapy

Level of sedation = assessed using either the Richmond Agitation-Sedation Scale (RASS) or the Riker Sedation-Agitation Scale (SAS)[17, 18], coma = RASS = -4 or-5 or Riker-SAS =1 or 2

SOFA= the Sequential Organ Failure Assessment score [13]

APACHE-II= the Acute Physiology and Chronic Health Evaluation score [12]

LOS-ICU= length of stay in the intensive care unit
