# Supplementary material for: Delirium prediction in the intensive care unit: comparison of two delirium prediction models
Source: Crit Care. 2018 May 5;22:114. doi: 10.1186/s13054-018-2037-6 (PMC5935943; doi:10.1186/s13054-018-2037-6)
Supplement: Supplementary file 3 — Table S2. Outcome user convenience questionnaire for ICU physicians. (DOCX 19 kb) [file 13054_2018_2037_MOESM3_ESM.docx]

**Additional file 3:Table S2** Outcome user convenience questionnaire for ICU physicians

| **Question** | **Score** | **E-PRE-DELIRIC**  **% of ICU physicians** | **PRE-DELIRIC**  **% of ICU physicians** |
| --- | --- | --- | --- |
| Time and effort needed to collect data to calculate a patient’s risk | Very low | 4.5 | 6.2 |
|  | Low | 37.9 | 27.7 |
|  | Neutral | 40.9 | 40.0 |
|  | High | 15.2 | 20.0 |
|  | Very high | 1.5 | 6.2 |
|  | | | |
| Burden for the physician to collect data about the predictors to calculate a patient’s risk | Very low | 1.5 | 1.5 |
|  | Low | 36.4 | 24.6 |
|  | Neutral | 37.9 | 36.9 |
|  | High | 21.2 | 29.2 |
|  | Very high | 3.0 | 7.7 |
|  | | | |
| Availability of predictors | Never available | 3.0 | 6.2 |
|  | Unavailable | 1.5 | 6.2 |
|  | Neutral | 24.2 | 23.1 |
|  | Available | 59.1 | 58.5 |
|  | Always available | 12.1 | 6.2 |
|  | | | |
| Clearness of the definitions of the predictors | Very vague | 1.5 | 1.5 |
|  | Vague | 3.0 | 1.5 |
|  | Neutral | 18.2 | 21.5 |
|  | Clear | 69.7 | 67.7 |
|  | Very clear | 7.6 | 7.7 |
|  | | | |
| Reliability of the outcome (predicted risk) of the prediction model | Very unreliable | 1.5 | 1.5 |
|  | Unreliable | 1.5 | 3.1 |
|  | Neutral | 68.2 | 69.2 |
|  | Reliable | 27.3 | 23.1 |
|  | Very reliable | 1.5 | 3.1 |
|  | | | |
| Are you going to use the delirium prediction model in daily practice | Not | 6.1 | 12.3 |
|  | Probably not | 31.8 | 30.8 |
|  | Neutral | 22.7 | 24.6 |
|  | Probably | 24.2 | 18.5 |
|  | Sure | 9.1 | 7.7 |
|  | Depends on this study | 6.1 | 6.2 |
|  | | | |
