# Supplementary material for: Delirium prediction in the intensive care unit: comparison of two delirium prediction models
Source: Crit Care. 2018 May 5;22:114. doi: 10.1186/s13054-018-2037-6 (PMC5935943; doi:10.1186/s13054-018-2037-6)
Supplement: Supplementary file 4 — Table S3. Quality check delirium assessment. (DOCX 18 kb) [file 13054_2018_2037_MOESM4_ESM.docx]

**Additional file 4:Table S3^#^** Quality check delirium assessment

| **Center** | **Inter-rater reliability**  **(mean Cohen’s kappa)** | **CAM-ICU or ICDSC compliance**  **(mean in %)** |
| --- | --- | --- |
| 10 'University Medical Centre Utrecht (UMU)' | 0.50 | 82 |
| 20 'Jeroen Bosch Ziekenhuis (JBZ)' | 1.00 | 100 |
| 40 'Antwerp University Hospital (AUH)' | * | 89 |
| 50 'The Canberra Hospital (TCH)' | 0.69 | 74 |
| 60 'Medisch Spectrum Twente (MST)' | 0.60 | 100 |
| 80 'Hospital Espírito Santo (HES)' | 1.00 | 86 |
| 90 'Erasmus MC (EMC)' | 1.00 | 95 |
| 10 'Tufts MC (TMC)' | 0.84 | 85 |
| 11 'Radboudumc (RMC)' | 0.60 | 91 |
| 12 'Rigshospitalet (RHL)' | 1.00 | 87 |
| 13 'Mt Sinai Hospital/U of Toronto (SHT)' | * | * |

*Not available

^#^Four ICUs were not able to perform the quality check of their delirium assessment completely according to the description in the method section
